# Constructing FeNiPt@C Trifunctional Catalyst by High Spin‐Induced Water Oxidation Activity for Zn‐Air Battery and Anion Exchange Membrane Water Electrolyzer

**DOI:** 10.1002/advs.202308205

**Published:** 2024-03-14

**Authors:** Yangdan Pan, Yuwen Li, Adeela Nairan, Usman Khan, Yan Hu, Baoxin Wu, Lu Sun, Lin Zeng, Junkuo Gao

**Affiliations:** ^1^ Institute of Functional Porous Materials The Key Laboratory of Advanced Textile Materials and Manufacturing Technology of Ministry of Education School of Materials Science and Engineering Zhejiang Sci‐Tech University Hangzhou 310018 China; ^2^ State Key Laboratory of Silicon Materials School of Materials Science and Engineering Zhejiang University Hangzhou 310058 China; ^3^ Department of Mechanical and Energy Engineering Southern University of Science and Technology Shenzhen 518055 China; ^4^ Institute of Modern Optics Tianjin Key Laboratory of Micro‐scale Optical Information Science and Technology Nankai University Tianjin 300350 China

**Keywords:** anion exchange membrane water electrolyzer, metal‐organic framework, trifunctional catalyst, water oxidation activity, Zn‐air battery

## Abstract

Developing cost‐efficient trifunctional catalysts capable of facilitating hydrogen evolution reaction (HER), oxygen evolution reaction (OER), and oxygen reduction reaction (ORR) activity is essential for the progression of energy devices. Engineering these catalysts to optimize their active sites and integrate them into a cohesive system presents a significant challenge. This study introduces a nanoflower (NFs)‐like carbon‐encapsulated FeNiPt nanoalloy catalyst (FeNiPt@C NFs), synthesized by substituting Co^2+^ ions with high‐spin Fe^2+^ ions in Hofmann‐type metal‐organic framework, followed by carbonization and pickling processes. The FeNiPt@C NFs catalyst, characterized by its nitrogen‐doped carbon‐encapsulated metal alloy structure and phase‐segregated FeNiPt alloy with slight surface oxidization, exhibits excellent trifunctional catalytic performance. This is evidenced by its activities in HER (−25 mV at 10 mA cm^−2^), ORR (half‐wave potential of 0.93 V), and OER (294 mV at 10 mA cm^−2^), with the enhanced water oxidation activity attributed to the high‐spin state of the Fe element. Consequently, the Zn‐air battery and anion exchange membrane water electrolyzer assembled by FeNiPt@C NFs catalyst demonstrate remarkable power density (168 mW cm^−2^) and industrial‐scale current density (698 mA cm^−2^ at 1.85 V), respectively. This innovative integration of multifunctional catalytic sites paves the way for the advancement of sustainable energy systems.

## Graphical abstract

The nanoflower‐like carbon‐encapsulated FeNiPt nanoalloy hierarchical structure (FeNiPt@C NFs catalyst) is successfully synthesized by pyrolyzing metal‐organic framework, exhibiting exceptional performance in HER/OER/ORR. Notably, spin‐induced enhanced water oxidation is observed for the first time in a carbon‐encapsulated alloy catalyst. Therefore, a trifunctional FeNiPt@C NFs catalyst is developed as an exemplary model for designing and synthesizing efficient and stable multifunctional catalysts.

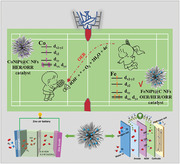

## 1 Introduction

The imperative shift from fossil fuels to renewable energy sources marks a crucial stride towards mitigating carbon emissions and fostering sustainable production and lifestyles. This transition is pivotal for the evolution of renewable energy storage and conversion technologies, notably anion exchange membrane water electrolyzer (AEMWE)^[^ ^1^, ^2^, ^3^ ^]^ and Zn‐air battery (ZAB),^[^ ^4^, ^5^, ^6^ ^]^ which are central to the oxygen evolution reaction (OER), hydrogen evolution reaction (HER), and oxygen reduction reaction (ORR). AEMWE technology is an environmentally friendly and pollution‐free chemical energy storage system that converts electrical energy into high‐energy‐density hydrogen gas. However, its success hinges on the development of affordable, durable bifunctional OER/HER electrocatalysts that boast high efficiency. Similarly, ZAB holds promising potential as a power source for renewable energy storage and electric vehicles, attributed to their safety, cost‐effectiveness, and high theoretical energy density.^[^ ^7^, ^8^ ^]^ Nevertheless, the efficiency of ZAB is hampered by the sluggish kinetics of OER and ORR during charge and discharge cycles at the air electrode,^[^ ^9^ ^]^ underscoring the necessity for high‐performance, cost‐efficient bifunctional OER/ORR electrocatalysts to propel ZAB technology forward. Enhancements in catalytic activity, stability, and affordability are crucial for advancing these energy devices, particularly through the simultaneous integration of multiple efficient catalytic sites within a single system.

Metal‐organic frameworks (MOFs) emerge as promising precursors for carbon‐based catalysts, customizable with precision to embed various catalytic active sites, thus ideal for crafting multifunctional catalysts.^[^ ^10^, ^11^, ^12^, ^13^ ^]^ Their inherent compatibility with diverse metal ions enables the creation of highly efficient active sites tailored for multifunctional catalysis.^[^ ^14^ ^]^ Furthermore, the N and O‐rich organic ligands in MOFs facilitate in situ heteroatom doping, enhancing electron transport and electrical conductivity of carbon species.^[^ ^15^, ^16^ ^]^ Despite these advantages, the exploration of MOF‐derived carbon‐encapsulated metal alloy trifunctional catalysts remains limited, and achieving optimal catalytic activity in all aspects of ORR/OER/HER is challenging. Notably, platinum (Pt)‐based alloy catalysts are esteemed for their optimal HER and ORR performance, attributed to their suitable adsorption energy for reaction intermediates (*H for HER, *O, *OOH, and *OH for ORR), which effectively promotes the kinetics of these reactions.^[^ ^17^, ^18^ ^]^ Additionally, Pt‐based alloy significantly increases catalytic activity compared to single metal by affecting the physicochemical properties (geometry/electronic property and coordination environment), resulting in the strain/ligand/ensemble effect. However, their OER performance typically falls short, prompting efforts to bolster OER activity without compromising ORR and HER efficacy.

Recent research into the spin‐dependent engineering of OER has shown promise.^[^ ^19^, ^20^, ^21^ ^]^ For instance, the incorporation of high‐spin elements in spinel oxides can modify the rate‐determining step during the OER process, thus enhancing its performance.^[^ ^22^, ^23^ ^]^ Expanding upon this concept, high‐spin Fe^2+^ ions were substituted for Co^2+^ ions in nanoflower (NFs)‐like CoNiPt‐MOF precursor based on the HER and ORR activities of CoNiPt@C catalyst.^[^ ^24^, ^25^ ^]^ Post‐pyrolysis and acid treatment yielded an advanced FeNiPt@C NFs catalyst, featuring a nitrogen‐doped carbon‐encapsulated FeNiPt nanoalloy with phase segregation. This configuration not only boosts carbon conductivity through charge transfer but also protects the catalytic sites from electrolyte erosion. Consequently, the FeNiPt@C NFs catalyst exhibits exceptional trifunctional catalytic performance, with a significant half‐wave potential of 0.93 V for ORR, and low overpotentials of 25 mV and 294 mV at 10 mA cm^−2^ for HER and OER, respectively. This outstanding performance can be attributed to the synergistic interaction between nitrogen‐doped carbon‐encapsulated metal alloy structure and phase‐segregated FeNiPt alloy with slight surface oxidization. Besides, the high spin state of the Fe element plays a crucial role in enhancing OER performance. Simultaneously, the exceptional OER/HER/ORR activity is also reflected in the ZAB and AEMWE assembled by the FeNiPt@C NFs catalyst. This new idea of integrating multiple catalytic active components within a single system presents a broad avenue for the advancement of sustainable energy devices.

## 2 Results and Discussion

### 2.1 Structural Characterizations of Catalysts

**Figure 1:**
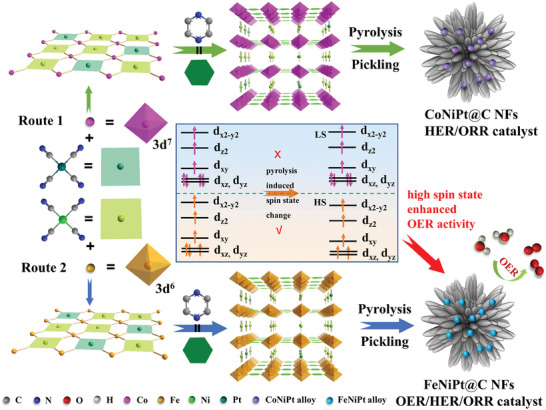
Synthetic schematic illustration of the FeNiPt@C NFs and CoNiPt@C NFs catalyst.

**Figure 2:**
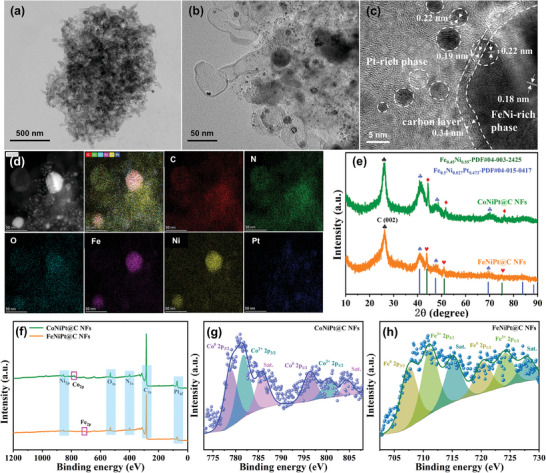
a,b) TEM images of the FeNiPt@C NFs. c) HRTEM image of the FeNiPt@C NFs. d) EDS elemental mapping of the FeNiPt@C NFs. e) PXRD spectrum of catalysts. f) XPS survey spectrum of catalysts. g) High‐resolution Co 2p XPS spectrum of CoNiPt@C NFs. h) High‐resolution Fe 2p XPS spectrum of the FeNiPt@C NFs.

The synthesis of trifunctional FeNiPt@C NFs catalyst involved carbonizing a trimetallic Hofmann‐type FeNiPt‐MOF precursor and subsequent acid impregnation to treat pyrolyzed particles, as illustrated in **Figure** **1**. Scanning electron microscopy (SEM) analysis (Figures S1 and S2, Supporting Information) reveals that both FeNiPt‐MOF and CoNiPt‐MOF precursors exhibit a nanoflower‐like morphology with thick sheets. This structure is preserved in the FeNiPt@C NFs and CoNiPt@C NFs catalysts post‐carbonization and pickling, with the emergence of interconnected short carbon nanotubes (CNTs) on their surfaces. Transmission electron microscopy (TEM) observations further confirm the formation of a 3D hierarchical structure within FeNiPt@C NFs catalyst, characterized by intertwined short CNTs (**Figure** **2**a). A multitude of nanoparticles with diverse sizes are randomly distributed throughout the carbon matrix (Figure 2b). The high‐resolution TEM (HRTEM) image (Figure 2c) clearly showcases a graphitic carbon layer (PDF#41‐1487) with lattice fringe spacing of 0.34 nm corresponding to the (002) crystal plane. The relatively small nanoparticles display a diffraction fringe spacing of 0.19 and 0.22 nm, corresponding to the (200) and (111) crystal planes of Fe_0.5_Ni_0.027_Pt_0.473_ (PDF#04‐015‐0417), indicating the formation of a Pt‐rich FeNiPt alloy phase. In contrast, the diffraction fringe spacing of relatively large nanoparticles is 0.18 nm, which is identified as the (200) plane of Fe_0.45_Ni_0.55_ (PDF#04‐003‐2425), suggesting the formation of a FeNi‐rich FeNiPt alloy phase. Figure 2d and Figure S3 (Supporting Information) illustrate the presence of the C, N, O, Fe, Ni, and Pt elements in FeNiPt@C NFs catalyst through TEM energy‐dispersive X‐ray spectra (EDS) elemental mapping and line scan results, respectively. Furthermore, the inhomogeneity of Fe, Ni, and Pt elements also indicates the existence of phase segregation in the FeNiPt alloy, which further validates the HRTEM results.

The powder X‐ray diffraction (PXRD) patterns (Figure S4, Supporting Information) indicate that the diffraction peaks of the as‐obtained FeNiPt‐MOF precursor are similar to those of CoNiPt‐MOF precursor and are well in agreement with the simulated CoNi‐MOF, suggesting successful synthesis of Hofmann‐typed MOF's unique column laminar frame structure. Following pyrolysis and pickling of the MOF precursor, the PXRD pattern of as‐synthesized FeNiPt@C NFs also demonstrates the formation of FeNi‐rich phase, Pt‐rich phase, and graphitic carbon (Figure 2e). The strong diffraction peak observed at 26.3° is attributed to the (002) plane of graphitic carbon,^[^ ^26^ ^]^ while the broad peaks at 43.8°, 51.1°, and 75.1° corresponded to the (111), (200), (220) plane of Fe_0.45_Ni_0.55_, indicating the formation of FeNi‐rich phase. Additionally, other peaks identified at 40.8°, 47.5°, 69.4°, and 83.7° are ascribed to the (111), (200), (220), (311) planes of Fe_0.5_Ni_0.027_Pt_0.473_, corresponding to the formation of Pt‐rich phase. These XRD results are consistent with HR‐TEM images and EDS mappings. The chemical composition and surface valence state of the catalysts were analyzed by X‐ray photoelectron spectroscopy (XPS). The XPS survey spectrum of FeNiPt@C NFs catalyst exhibits distinct peaks corresponding to C, N, O, Fe, Ni, and Pt elements (Figure 2f), which further confirms the successful synthesis of ternary FeNiPt alloy nanoparticles. The high‐resolution N 1s spectra observe three distinct fitting peaks (Figure S5, Supporting Information), assigning to pyridinic N (398.3 eV), pyrrolic N (400.4 eV), and graphitic N (401.9 eV).^[^ ^27^, ^28^ ^]^ Table S1 (Supporting Information) summarizes the proportions of different N species in the catalysts. For FeNiPt@C NFs catalyst, pyridinic N, pyrrolic N, and graphitic N account for 50.1%, 35.4%, and 14.4%, respectively. The CoNiPt@C NFs catalyst exhibits similar proportions of N species. The O 1s spectra are deconvolved into three typical O species, including ‐O‐metal (530.9 eV), defects O (532.3 eV), and surface OH^−^/O_2_ (533.1 eV) (Figure S6, Supporting Information).^[^ ^29^, ^30^, ^31^ ^]^ Both FeNiPt@C NFs and CoNiPt@C NFs catalysts exhibit similar proportions of different O species, with the highest proportion being that of O defects (Table S2, Supporting Information). Moreover, the high‐resolution Co 2p spectrum of CoNiPt@C NFs shows two prominent fitting peaks at 778.2 eV (Co 2p_3/2_) and 795.5 eV (Co 2p_1/2_), which are attributed to metallic Co. The minor peaks at 780.9 and 799.7 eV can be assigned to Co^2+^ in the form of Co^2+^ 2p_3/2_ and Co^2+^ 2p_1/2_, accompanied by two satellite peaks located at 785.1 eV and 803.6 eV (Figure 2g).^[^ ^32^, ^33^, ^34^ ^]^ Similarly, the high‐resolution Fe 2p spectrum (Figure 2h) exhibits three pairs of double peaks corresponding to the Fe^0^, Fe^3+,^ and satellite peaks. The characteristic peaks at 707.5 eV and 720.3 eV are attributed to Fe 2p3/2 and Fe 2p1/2 energy levels of Fe^0^, while those located at 711.1 and 724.1 eV are assigned to Fe 2p3/2 and Fe 2p1/2 of Fe^3+^.^[^ ^35^, ^36^ ^]^ The remaining peaks observed at 715.1 and 727.9 eV are attributed to satellite peaks. These results demonstrate the presence of a metallic state and partially oxidized state in Co and Fe elements on the surface of the catalysts. The oxidation state of the CoNiPt@C NFs catalyst is divalent, while that of the FeNiPt@C NFs catalyst is trivalent. The modulation of electronic states induced by different metal components can result in diverse electrocatalytic OER performance, as inferred. The high‐resolution Ni 2p spectra of both FeNiPt@C NFs and CoNiPt@C NFs sample (Figure S7, Supporting Information) exhibit three pairs of bimodal peaks corresponding to the Ni^0^, Ni^2+^, and satellites peaks.^[^ ^37^ ^]^ The high‐resolution Pt 4f spectra of FeNiPt@C NF and CoNiPt@C NFs display two pairs of peaks at 71.4/74.6 and 72.4/75.6 eV, corresponding to Pt^0^ 4f_7/2_ / Pt^0^ 4f_5/2_, and Pt^2+^ 4f_7/2_ / Pt^2+^ 4f_5/2_, respectively (Figure S8, Supporting Information).^[^ ^38^ ^]^ For both Ni and Pt elements in catalysts, the peak height of the metal state is higher than that of the oxidized state, indicating that the metal state predominates in these two alloy elements.

### 2.2 Synchrotron Radiation X‐Ray Analysis

**Figure 3:**
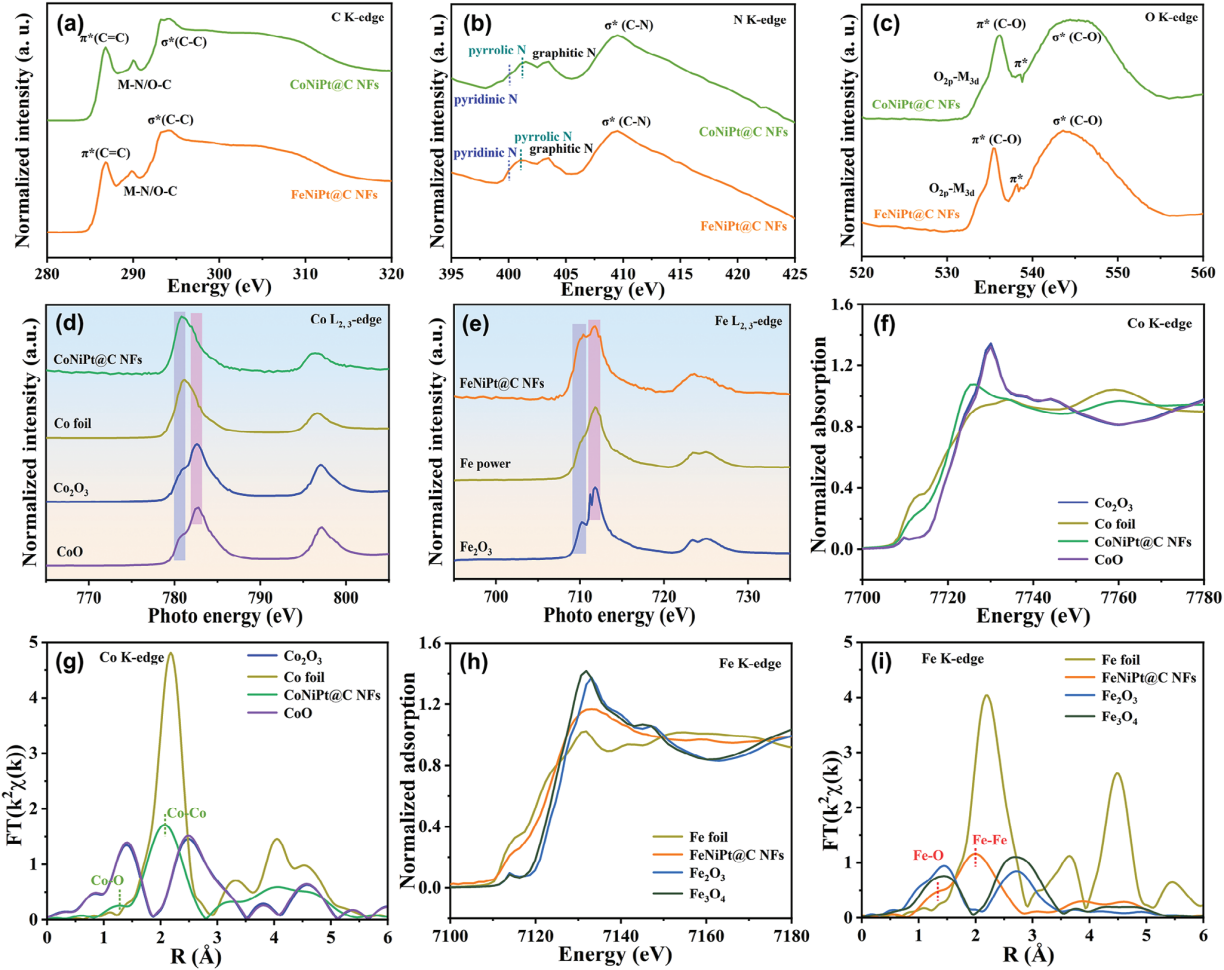
a) C K‐edge XANES spectra. b) N K‐edge XANES spectra. c) O K‐edge XANES spectra. d) Co L_2,3_‐edge XANES spectra. e) Fe L_2,3_‐edge XANES spectra. f) Co K‐edge XANES spectra of CoNiPt@C NFs and standard sample. g) Corresponding k^2^‐weight FT‐EXAFS spectra of Co K‐edge. h) Fe K‐edge XANES spectra of the FeNiPt@C NFs and standard sample. i) Corresponding k^2^‐weight FT‐EXAFS spectra of Fe K‐edge.

Soft X‐ray absorption near edge structure (XANES) spectroscopy provided insights into the local electronic structure, including valence state, spin, and symmetry of the metal atom within the catalysts. As shown in **Figure** **3**a, the C K‐edge XANES spectra reveal three obvious characteristic peaks: π^*^_C═C_, π^*^_M─N/O─C,_ and σ^*^_C─C_. The presence of π^*^_C═C_ and σ^*^_C─C_ indicates a highly conductive sp^2^‐hybridized carbon.^[^ ^39^ ^]^ While the minor peak of π^*^_M─N/O─C_ at 288.8 eV suggests the occurrence of a chemical interaction between a metal element and carbon substrate, which can effectively facilitate catalysis by promoting rapid electron transfer.^[^ ^40^ ^]^ Moreover, the N K‐edge XANES spectra display four distinct spectroscopic features corresponding to pyridinic N, pyrrolic N, graphitic N, and σ^*^_C‐N_ (Figure 3b)_,_^[^ ^41^, ^42^ ^]^ aligning with high‐resolution N 1s spectral findings. The O K‐edge XANES spectra exhibit four distinctive peaks, namely ‐O_2p_‐M_3d_, π* (C‐O), π*, and σ* (C─O) (Figure 3c).^[^ ^43^, ^44^, ^45^ ^]^ Figure 3d illustrates the normalized Co L_2,3_‐edge spectra of CoNiPt@C NFs catalyst in comparison with CoO, Co_2_O_3_, and Co foil. The spectral features are primarily governed by the spin‐orbit coupling of Co 2p core‐hole, resulting in a dichotomy of the spectrum into two distinct regions: L_3_ (782 eV) and L_2_ (796 eV). It was discovered that cobalt element within CoNiPt nanoparticles predominantly exists in a metallic state, with only minor amounts of surface‐bound Co^2+^ ions present. The normalized Fe L_2,3_‐edge spectra of FeNiPt@C NFs catalyst and reference samples (Fe_2_O_3_ and Fe powder) are presented in Figure 3e. The absorption spectrum (711 eV) at the Fe L_3_‐edge originates from the transition between Fe 2p and 3d orbitals. The characteristic absorption spectrum of the FeNiPt@C NFs catalyst resembles those of the Fe_2_O_3_ sample, indicating that high‐spin Fe^3+^ within FeNiPt nanoparticles is formed. Likewise, the standardized Ni L_2,3_‐edge XANES profiles reveal discernible peaks at 873 and 856 eV indicative of Ni L_2_ (from 2p_1/2_→3d) and Ni L_3_ (from 2p_3/2_→3d) transitions, respectively (Figure S9, Supporting Information).^[^ ^46^, ^47^ ^]^ In addition, hard X‐ray absorption techniques, including XANES and extended X‐ray absorption fine structure (EXAFS), elucidated the catalysts’ local electronic structures and coordination environment. The normalized Co K‐edge XANES spectra reveal that the pre‐edge peak of CoNiPt@C NFs is situated between that of the metallic Co foil and oxidized CoO, suggesting a slightly oxidized valence state for the Co element (Figure 3f). The corresponding Co K‐edge EXAFS spectra exhibit a discernible scattering peak at 2.1 Å (without phase correction) originating from Co─Co/Ni/Pt configuration,^[^ ^48^ ^]^ and a minor scattering peak at 1.4 Å arising from the Co─O configuration (Figure 3g).^[^ ^49^ ^]^ Similarly, the normalized Fe K‐edge XANES spectra of FeNiPt@C NFs catalyst exhibit a comparable trend as described above. The pre‐edge energy of FeNiPt@C NFs is situated between that of Fe foil and Fe_2_O_3_, indicating the oxidation state of the Fe element (Figure 3h). The Fe K‐edge EXAFS spectra demonstrate two notable scattering peaks, which can be ascribed to the contribution of Fe─O and Fe‐Fe/Ni/Pt with distances of 1.3 and 2.1 Å, respectively (Figure 3i).^[^ ^50^, ^51^, ^52^ ^]^ The normalized Pt L_3_‐edge XANES spectra suggest that the valence state of Pt in both FeNiPt@C NFs and CoNiPt@C NFs catalysts is predominantly metallic, as indicated by the positioning of the white‐line peak between that of commercial Pt/C catalyst and Pt foil (Figure S10, Supporting Information). Besides, similar scattering peaks at 2.2 Å (no phase shift) are observed in the FT k^2^‐weighted Pt L_3_‐edge EXAFS spectra, which can be attributed to the Pt─M (M = Pt, Ni, Co, or Fe) coordination.^[^ ^53^, ^54^ ^]^

### 2.3 Alkaline OER/HER/ORR Performance Evaluation on a RDE Set‐Up

**Figure 4:**
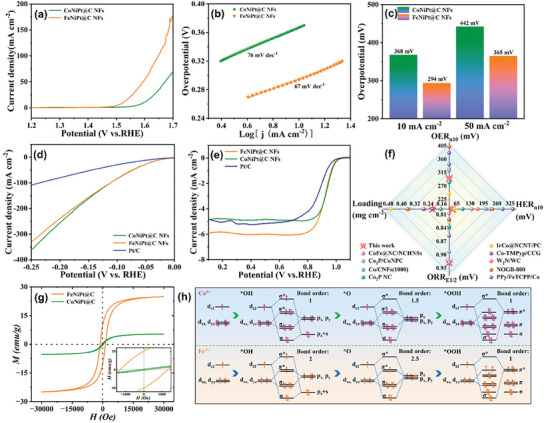
a) Polarization curves for alkaline OER at 1600 rpm. b) The corresponding Tafel plots. c) The corresponding overpotential. d) Polarization curves for alkaline HER at 1600 rpm. e) Polarization curves for alkaline ORR at 1600 rpm. f) Radar map of catalytic performance (OER_n10_, HER_n10_, ORR_E1/2_) and loading. g) Magnetic hysteresis loops of CoNiPt@C NFs and FeNiPt@C NFs at 300 K temperature, inset: the enlarged H_c_ and B_r_ region. h) Schematic illustration of a possible mechanism of enhanced OER activity (the orbital interaction between the active site and the OER intermediates).

The electrocatalytic OER/HER/ORR activities of FeNiPt@C NFs and CoNiPt@C NFs catalysts were evaluated by a rotating disk electrode (RDE) set up in an alkaline medium, detailed in the experimental section (Supporting Information). **Figure** **4**a plots the OER linear sweep voltammograms of the catalysts to explore the spin state‐dependent alkaline OER activity. Compared with CoNiPt@C NFs counterpart, the FeNiPt@C NFs catalyst demonstrates significantly enhanced OER catalytic performance, attributed to the high‐spin state of Fe^3+^, as indicated by a lower Tafel slope of 67 mV dec^−1^, suggesting faster reaction kinetics (Figure 4b). This is supported by a Nyquist plot showing a smaller semicircle radius for FeNiPt@C NFs catalyst, denoting high electronic conductivity (Figure S11 and Table S3, Supporting Information). Notably, the FeNiPt@C NFs catalyst exhibits lower overpotentials of 294 and 365 mV at 10 and 50 mA cm^−2^ current density, indicative of improved catalytic efficiency compared to CoNiPt@C NFs catalyst (Figure 4c). The OER chronopotentiometric curve at 10 mA cm^−2^ current density for FeNiPt@C NFs catalyst as shown in Figure S12 (Supporting Information), suggests excellent long‐term OER stability. For alkaline HER, both catalysts show similar performance with a low overpotential of 25 mV at 10 mA cm^−2^ current density, suggesting the spin state's minimal impact on alkaline HER activity (Figure 4d). Similarly, both catalysts exhibit an identical half‐wave potential (E_1/2_ = 0.93 V) in alkaline ORR polarization curves, while differences in limiting current density are attributed to variations in sample dispersion uniformity on the RDE (Figure 4e). This underscores the comparable efficiency of FeNiPt@C NFs and CoNiPt@C NFs catalysts in alkaline ORR, indicating that the alkaline ORR activity in this system is independent of the high spin state. Catalyst loading, overpotential at 10 mA cm^−2^ (n_10_), and half‐wave potential (E_1/2_) are key evaluation indices for catalyst cost and reaction efficiency. Comparative analysis with reported trifunctional catalysts emphasizes FeNiPt@C NFs catalyst's optimal performance across critical evaluation indices, suggesting its scalability for large‐scale applications (Figure 4f; Table S4, Supporting Information). According to the aforementioned valence state characterizations, the Fe element in FeNiPt@C NFs with a trivalent state possesses a greater number of spin electrons than the Co element in CoNiPt@C NFs with a divalent state. The hysteresis loops determined by Quantum Design PPMS‐9 at 300 K further reveal both catalysts’ ferromagnetism (FM) properties (Figure 4g). However, the FeNiPt@C NFs catalyst exhibits superior magnetic characteristics with coercivity (H_c_) and remanence (B_r_) values of 1310 Oe and 10 emu g^−1^, respectively, compared to those of the CoNiPt@C NFs catalyst (210 Oe and 0.288 emu g^−1^). This suggests that electrons in FeNiPt@C NFs are arranged in a high‐spin configuration. Combined with the electrochemical results, a robust ferromagnetic catalyst containing high electron spin state elements can significantly enhance the alkaline OER activity. Currently, the theoretical comprehension of the electrocatalytic OER mechanism encompasses not only the thermodynamic characteristics of reactant and intermediate adsorption/dissociation but also electron transfer kinetics and orbital interactions.^[^ ^55^, ^56^ ^]^ Therefore, the potential molecular orbital interactions between Co^2+^ and Fe^3+^ on catalyst surfaces and intermediate species (^*^OH, ^*^O, ^*^OOH, where * denotes active site) were analyzed. Considering the principle of symmetry conservation, the orbital interactions between d_xy_ and d_x_^2^_‐y_^2^ with intermediates can be disregarded.^[^ ^57^ ^]^ The orbital interactions between d_z_^2^, d_xz_, and d_yz_ with the reaction intermediates are illustrated in Figure 4h. The bond order serves as a quantitative indicator of the strength of interaction between the metal cation on the catalyst surface and the reaction intermediate. It can be calculated by the formula (bond order = 1/2 (number of bonding electrons – number of antibonding electrons)). As widely acknowledged, ^*^OH acts as the initial intermediate in the OER reaction and its strong adsorption can efficiently capture the reactants to initiate the OER cycle. Conversely, ^*^OOH serves as the final intermediate in the OER reaction, and its weak adsorption facilitates the rapid release of oxygen products.^[^ ^58^ ^]^ Therefore, an efficient OER catalyst should exhibit strong adsorption of ^*^OH intermediates and weak adsorption of ^*^OOH intermediates during the alkaline OER catalytic progress. From the diagram, it is apparent that the bond order of ^*^OH or ^*^OOH intermediates on CoNiPt@C NFs catalyst is 1, whereas for FeNiPt@C NFs catalyst, the bond order between and ^*^OH or ^*^OOH intermediates is 2 and 1, respectively. Hence, the FeNiPt@C NFs catalyst demonstrates superior OER activity compared to the CoNiPt@C NFs catalyst, which is consistent with our OER performance test results. This innovative approach of incorporating high‐spin metal elements into the alloy has markedly improved alkaline OER performance, establishing FeNiPt@C NFs catalyst as an efficient and cost‐effective trifunctional catalyst suitable for energy conversion technologies.

### 2.4 ZAB and AEMWE Analysis

**Figure 5:**
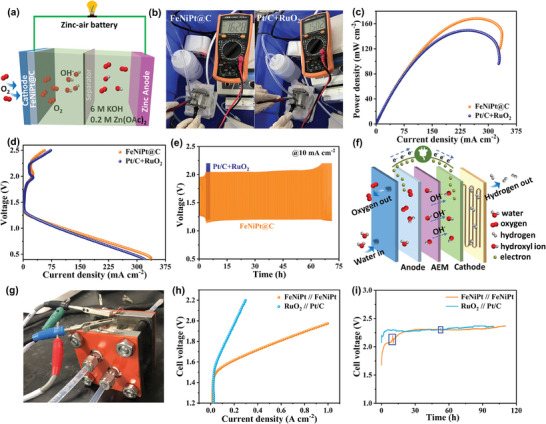
a) Schematic diagram of ZAB. b) The optical photograph of OCP in assembled FeNiPt@C ZAB. c) Peak power density curves. d) Charge and discharge polarization curves. e) Galvanostatic discharge–charge cycling curves at a current density of 10 mA cm^−2^. f) Schematic diagram of AEMWE. g) The optical photograph of AEMWE. h) Polarization curves (no *iR*‐compensation) of AEMWE at 60 °C. i) Chronopotentiometry curve at 200 mA cm^−2^.

The practical application of the trifunctional FeNiPt@C NFs catalyst was validated in two energy conversion devices: the ZAB and AEMWE, respectively. The FeNiPt@C NFs catalyst acts as a bifunctional OER and ORR catalyst, integrated into the air electrode of the ZAB (**Figure** **5**a). For comparison, commercial Pt/C and RuO_2_ catalysts in a 1:1 mass ratio were employed to fabricate the air electrode as a reference. As demonstrated in Figure 5b, the open circuit potential (OCP) of the FeNiPt@C‐assembled ZAB was initially measured at 1.628 V, surpassing that of the commercial Pt/C+RuO_2_ ZAB (1.502 V). Additionally, the maximum power density of the FeNiPt@C ZAB reached 168 mW cm^−2^ (Figure 5c), outperforming that of Pt/C+RuO_2_ ZAB (149 mW cm^−2^). The charge‐discharge polarization curves illustrate that the charge‐discharge voltage gap of the FeNiPt@C ZAB is comparable to that observed in the Pt/C+RuO_2_ ZAB (Figure 5d). Furthermore, the FeNiPt@C‐based ZAB achieves a specific capacity of 851.66 A h kg_Zn_^−1^ and a high specific energy density of 1051.80 W h kg_Zn_^−1^ at 10 mA cm^−2^ (Figure S13, Supporting Information). The charge‐discharge cycling curve serves as a crucial indicator for evaluating the long‐term stability of ZABs, which was examined at a current density of 10 mA cm^−2^ with charging and discharging intervals set at 15 min. The Pt/C+RuO_2_ ZAB exhibits unsatisfactory charge‐discharge cycling behavior attributed to the inadequate OER stability of the RuO_2_ catalyst (Figure 5e), whereas the FeNiPt@C ZAB demonstrates a reduced voltage gap and enhanced round‐trip efficiency (58%). After the galvanostatic discharge–charge cycling test, the optical photographs, SEM, and XPS analysis in the FeNiPt@C ZAB were shown in Figures S14, S15, and S16 (Supporting Information), respectively. The presence of dendrites on the Zn plate is clearly observable, while the nanoflower‐like morphology of the FeNiPt@C NFs catalyst in the air electrode remains distinctive. Additionally, the high spin state of FeNiPt@C NFs catalyst also remains. The assembled FeNiPt@C ZAB not only exceeded the performance of the Pt/C+RuO_2_ ZAB but also showcased its potential for real‐world energy conversion applications. Furthermore, a FeNiPt@C AEMWE was constructed to verify the alkaline OER and HER activity of the FeNiPt@C NFs catalyst. As depicted in Figure 5f, the FeNiPt@C NFs catalyst as OER and HER catalyst was sprayed on the carbon paper (CP) to fabricate the anode and cathode of AEMWE, respectively. The membrane electrode assembly (MEA) was then constructed by sandwiching the FeNiPt@C/CP, commercial AEM, and another layer of FeNiPt@C/CP. Similarly, a RuO_2_ // Pt/C AEMWE was assembled by commercial RuO_2_/CP, AEM, and 20% Pt/C/CP as a reference, the optical photograph is shown in Figure 5g. The FeNiPt@C AEMWE demonstrates superior water electrolysis performance at 60 °C, achieving an industrial current density of 698 mA cm^−2^ at 1.85 V, a clear improvement over the RuO_2_ // Pt/C AEMWE (Figure 5h). Furthermore, the long‐term stability of the AEMWE was evaluated by subjecting it to continuous constant‐current electrolysis at a current density of 200 mA cm^−2^ (Figure 5i). Following an initial 20‐h electrolysis period, the cell potential of the FeNiPt@C AEMWE approached that of RuO_2_ // Pt/C AEMWE, increasing significantly from 1.67 to 2.26 V. Subsequently, the potential stabilized within a range of 2.26–2.36 V for a duration of 90 h, with a potential degradation rate of 1.11 mV h^−1^. The voltage fluctuations marked in blue rectangles are a result of electrolyte replenishment. Optical photographs and SEM images of the FeNiPt@C NFs catalyst in the cathode after stability measurement were illustrated in Figures S17 and S18 (Supporting Information), demonstrating that its nanoflower‐like structure remains intact. However, prolonged high current conditions cause fragility of the anode CP, resulting in close adhesion of the FeNiPt@C NFs catalyst to the AEM. These results confirm the FeNiPt@C NFs catalyst's suitability for scalable industrial applications, highlighting its structural durability and efficiency across both ZAB and AEMWE platforms.

## 3 Conclusion

In summary, this work introduces a novel approach to enhance OER catalytic performance through the strategic induction of higher spin states in metal ions within the MOF precursor, culminating in the development of carbon‐encapsulated FeNiPt alloy nanoparticles. The hierarchical nanoflower‐like the FeNiPt@C NFs catalyst exhibited outstanding trifunctional catalytic performance, highlighted by low overpotentials for HER (25 mV) and OER (294 mV) at a current density of 10 mA cm^−2^, and impressive half‐wave potential for ORR (0.93 V). This exceptional performance stems from the synergistic interaction between the nitrogen‐doped carbon‐encapsulated metal alloy structure and phase‐segregated FeNiPt active site with slight surface oxidation. Compared with the CoNiPt@C NFs counterpart with HER/ORR activity, the inclusion of a high‐spin Fe element notably enhanced the water oxidation activity the FeNiPt@C NFs catalyst. Additionally, the application of the FeNiPt@C NFs catalyst in energy conversion devices, including ZAB and AEMWE, demonstrated its practical effectiveness. The FeNiPt@C NFs catalyst‐assembled ZAB and AEMWE achieved significant power density (168 mW cm^−2^) and industrial‐level current density (698 mA cm^−2^ at 1.85 V), respectively. This study showcases the potential of integrating various catalytic active sites into a unified system, paving the way for advancing multifunctional catalysts in energy storage and conversion technologies.

## Conflict of Interest

The authors declare no conflict of interest.

## Supporting information

Supporting Information

## Data Availability

The data that support the findings of this study are available from the corresponding author upon reasonable request.
