# Supplementary material for: Constructing FeNiPt@C Trifunctional Catalyst by High Spin‐Induced Water Oxidation Activity for Zn‐Air Battery and Anion Exchange Membrane Water Electrolyzer
Source: Adv Sci (Weinh). 2024 Mar 14;11(19):2308205. doi: 10.1002/advs.202308205 (PMC11109642; doi:10.1002/advs.202308205)
Supplement: Supplementary file 1 — Supporting Information [file ADVS-11-2308205-s001.pdf]

ADVANCED SCIENCE
Open Access

Supporting Information

for *Adv. Sci.*, DOI 10.1002/advs.202308205

Constructing FeNiPt@C Trifunctional Catalyst by High Spin-Induced Water Oxidation Activity for Zn-Air Battery and Anion Exchange Membrane Water Electrolyzer

Yangdan Pan, Yuwen Li, Adeela Nairan, Usman Khan, Yan Hu, Baoxin Wu, Lu Sun, Lin Zeng and Junkuo Gao**

Supporting Information

Constructing FeNiPt@C Trifunctional Catalyst by High Spin-Induced OER Activity for Zn-air Battery and Anion Exchange Membrane Water Electrolyzer

Yangdan Pan, Yuwen Li, Adeela Nairan, Usman Khan, Yan Hu, Baoxin Wu, Lu Sun, Lin Zeng, Junkuo Gao**

Experimental Section

Synthesis of FeNiPt NFs MOF precursor. Solution A was prepared by dissolving 1.2 mmol of $Fe(ClO_4)_2{\cdot}xH_2O$ (304 mg), 1.2 mmol of Pyrazine (96 mg), and PVP (400 mg) in a water/methanol mixture solution (v:v=1:1) with a total volume of 160 mL. Separately, solution B was prepared by dissolving 1.08 mmol of $K_2[Ni(CN)_4]{\cdot}xH_2O$ (264 mg) and 0.12 mmol of $K_2[Pt(CN)_4]{\cdot}3H_2O$ (26 mg) in 40 mL DI water, which was then slowly added to solution A followed by continuous reaction for 3 hours. Upon completion of the reaction, the reactant was subjected to filtration and washed three times with a water/methanol mixture. The resulting orange precipitate was subsequently dried at 60 ℃ in an oven. Similarly, CoNiPt NFs MOF precursor was synthesized using the same procedure as described above, except that the $Fe(ClO_4)_2{\cdot}xH_2O$ was substituted with $Co(NO_3)_2{\cdot}6H_2O$ (348 mg).

Preparation of FeNiPt@C and CoNiPt@C catalysts. The synthesized MOF precursors were subjected to carbonization in a tube furnace under N_2 atmosphere, heated to 800 ℃ with a heating rate of 5 ℃ min^{-1} for 2 hours. Then, the resulting black powder was subjected to treatment with 3% HCl aqueous solution until effervescence ceased. Finally, the black precipitate was thoroughly washed with water and dried at a temperature of 60 ℃ in a drying oven.

Characterizations. The X-ray diffraction (XRD) data were collected using a Bruker AXS D8 Advance X-ray powder diffractometer equipped with a sealed Cu tube (λ=1.5406 Å). The X-ray photoelectron spectra (XPS) were acquired on Thermo Scientific K-Alpha and the binding energy was calibrated against the C_{1s} peak (284.8 eV). High-resolution TEM images were

obtained on an FEI TF20 transmission electron microscope, while EDS mapping images were generated on Super-X. The hard X-ray Absorption Fine Structure (XAFS) spectra were acquired in transmission mode at beamline BL11B located within the Shanghai Synchrotron Radiation Facility (SSRF). The soft X-ray Absorption Near-Edge Structure (XANES) spectra were gathered at beamline MCD-A situated in the National Synchrotron Radiation Laboratory (NSRL) in Hefei, China.

Electrochemical measurements. Electrocatalytic performance was conducted on a German Zahner electrochemical station with a traditional three-electrode system. The Hg/HgO and the graphite rod electrode were used as the reference and counter electrodes for HER and OER in 1.0 M KOH, while the Ag/AgCl and the Pt wire were used as reference and counter electrodes for ORR in 0.1 M KOH. The working electrode was a rotating disk electrode (RDE) (diameter: 5 mm, area: 0.19625 cm^{-2}). A catalyst ink (2 mg mL^{-1}) was prepared as follows: 2 mg of catalyst was dispersed in a mixture solution (960 uL IPA and 40 uL Nafion) under ultrasonication for 30 minutes. 25 uL of ink was dropped into the polished RDE electrode and air-dried at room temperature. The catalyst loading was controlled to be 0.254 mg cm^{-2}. The commercial Pt/C catalyst was used as the contrast sample, while the ink was prepared the same way as described above. Line scan voltammetry (LSV) curves were recorded at a scan rate of 5 mV s^{-1} for HER and OER measurements. The electrochemical impedance spectroscopy (EIS) for OER was conducted over a frequency range of 0.1 Hz to 100000 Hz with a bias voltage of 0.6 V and an amplitude of 10 mV. LSV curves were employed for ORR measurements at 20 mV s^{-1} scan rate. All LSV curves were corrected with 85% *iR*-compensation. The following equation determined the reversible hydrogen electrode potentials: E (V *vs.* RHE) = E (V *vs.* Hg/HgO) + 0.098 + 0.059 pH; E (V *vs.* RHE) = E (V *vs.* Ag/AgCl) + 0.197 + 0.059 pH.

Zn-Air Batteries assembly testing. The ZAB was constructed utilizing an air-electrode as the cathode and Zn plate (0.5 mm thick) as the anode, with a distance of 1.4 cm between them. The air electrode was fabricated by spraying OER/ORR catalyst ink onto the gas diffusion layer (diameter: 1 cm), resulting in a catalyst loading of 1.6 mg cm^{-2}. The catalyst ink was mixed with 5 mg FeNiPt NFs (or 20 wt% Pt/C+RuO_2, mass ratio 1:1) catalyst, 35 uL of 5 wt% Nafion, and 1 mL isopropanol. As a comparison sample, the loading of commercial Pt/C and RuO_2 catalyst was 1.64 mg cm^{-2}. The volume of ZAB was 12 mL and filled with 6.0 M KOH and 0.2 M $Zn(OAc)_2$ electrolyte. The performance of the assembled ZAB was collected on an electrochemical workstation (Shanghai Chenhua). The I-V curves were obtained by Linear Sweep Voltammetry with a scan rate of 5 mV s^{-1}. Additionally, the charge-discharge cycling

curves were examined by chronopotentiometry with a discharging time of 15 minutes and a charging time of 15 minutes at a current density of 10 mA cm^{-2}.

Water-alkali electrolysis assembly testing. A home-made AEMWE was assembled by two stainless-steel bipolar plates and a membrane electrode assembly (MEA). A single serpentine flow field, measuring 1.0 mm in width and 0.5 mm in depth with a rib of 1.0 mm, was precisely machined onto stainless-steel bipolar plates to create an area of 4 cm^2. The MEA was assembled by sandwiching the anode electrode, an anion exchange membrane (Tokuyama A901, 10 μm) and a cathode electrode. The catalyst ink was prepared by mixing FeNiPt@C NFs with 5 wt% Nafion in a mixture of deionized water and ethanol, followed by sonication for 30 minutes and spray-coating onto a carbon paper substrate (2 cm*2 cm). The catalyst loading was controlled at 2.0 mg cm^{-2} on each electrode. For comparison, the RuO_2//Pt/C AEMWE was assembled by the same process and catalyst loading. The anode catalyst consisted of commercial RuO_2, while the cathode catalyst was made up of commercial Pt/C. The performance evaluation of the AEMWE was conducted at 60 °C on an electrochemical workstation (Solartron 1470E). Both anode and cathode were supplied with 1.0 M KOH electrolyte by a peristaltic pump. All electrochemical data were measured after the cell performance was steady.

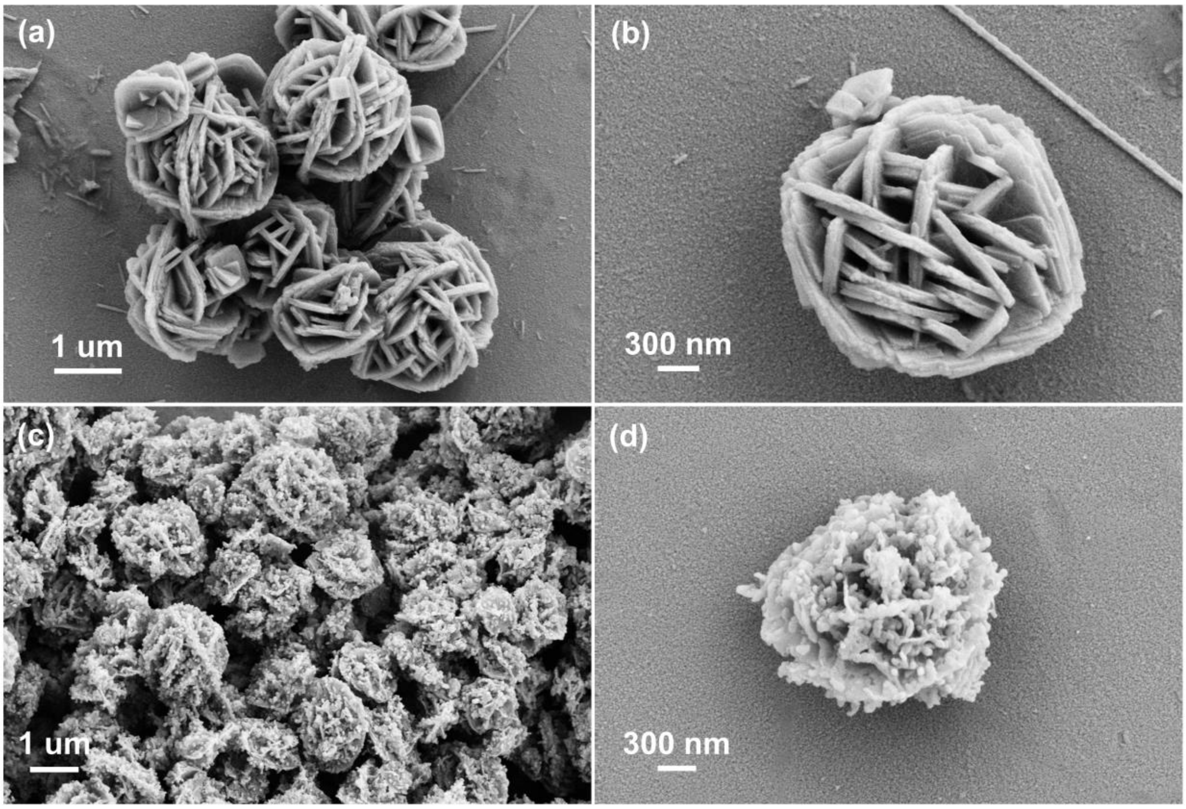

Figure S1. SEM images: (a-b) FeNiPt-MOF precursor. (c-d) FeNiPt@C NFs catalyst.

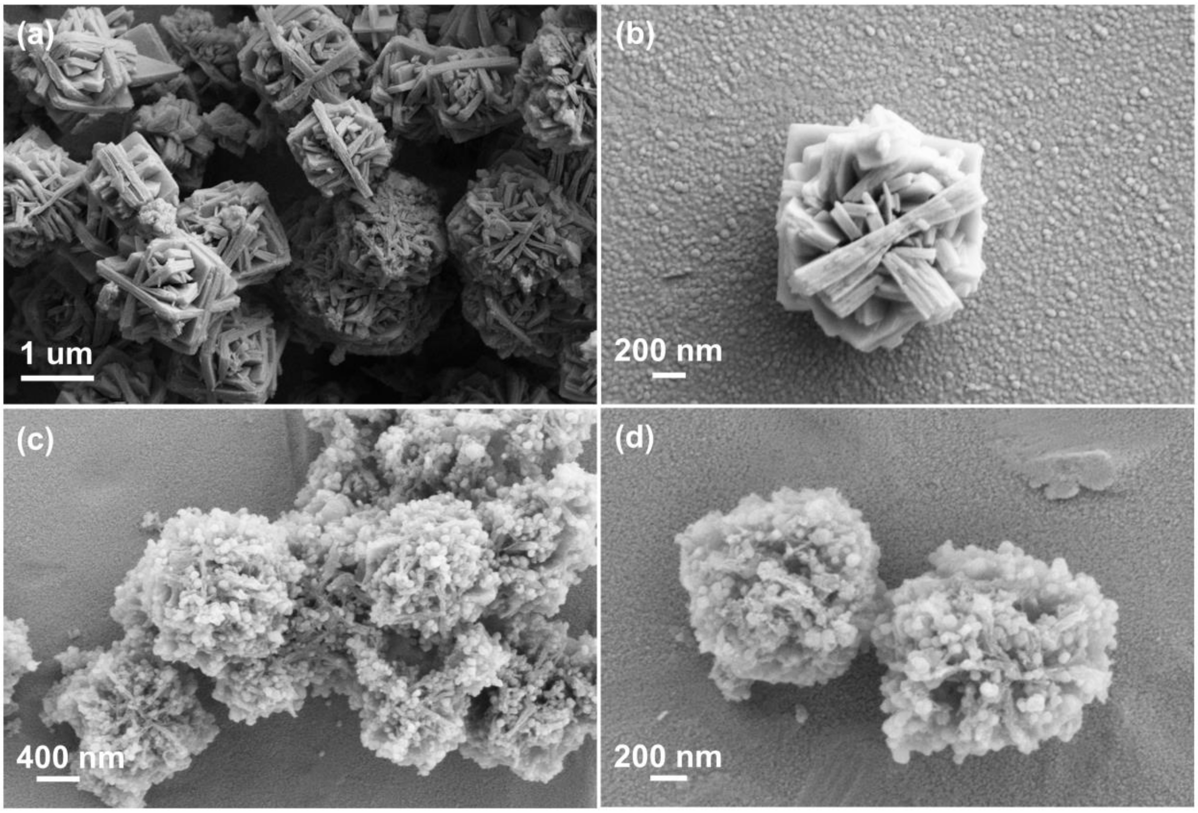

Figure S2. SEM images: (a-b) CoNiPt-MOF precursor. (c-d) CoNiPt@C NFs catalyst.

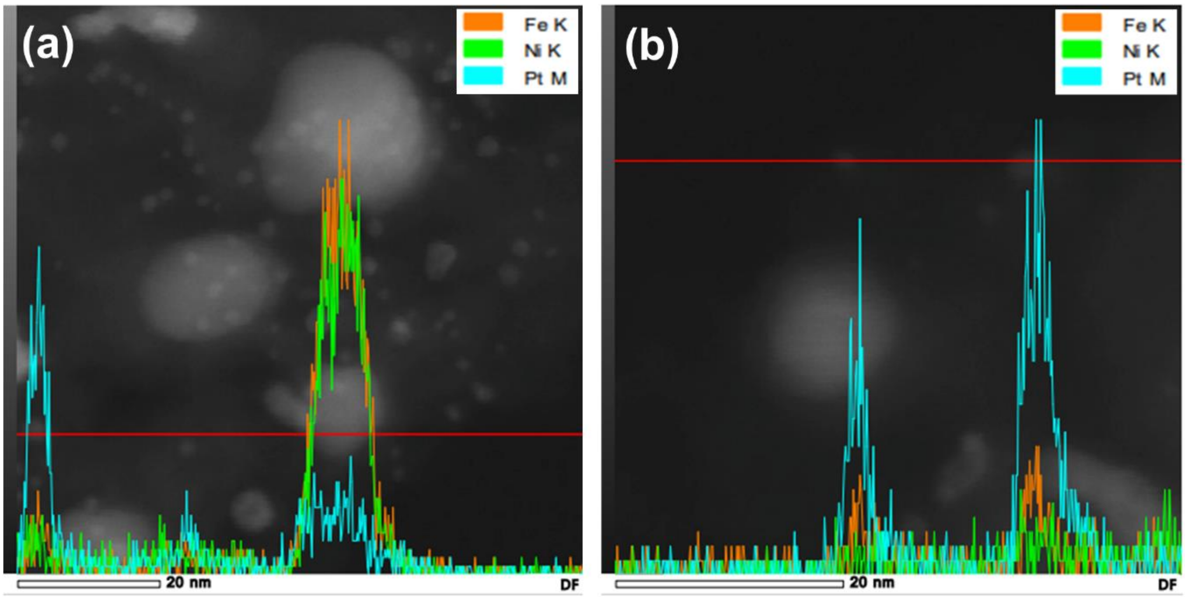

Figure S3. TEM line scanning results (a-b) of FeNiPt@C NFs catalyst in a different region.

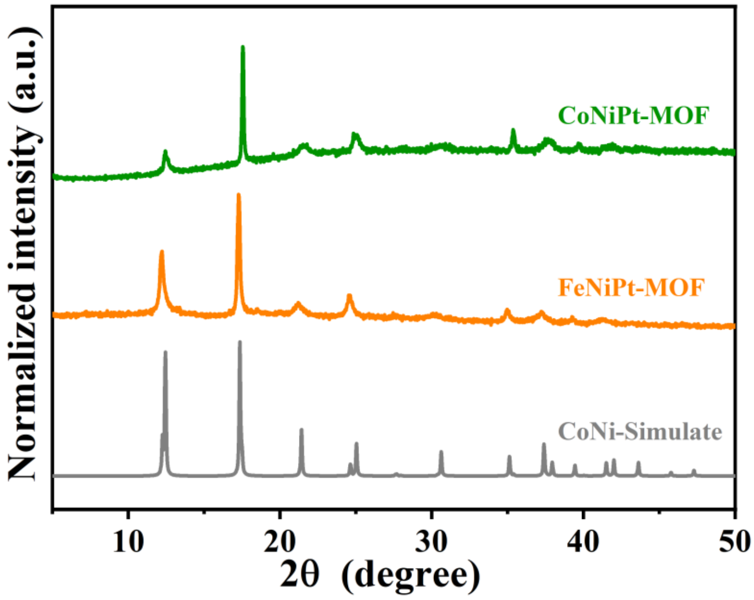

Figure S4. PXRD pattern of FeNiPt-MOF and CoNiPt-MOF precursors.

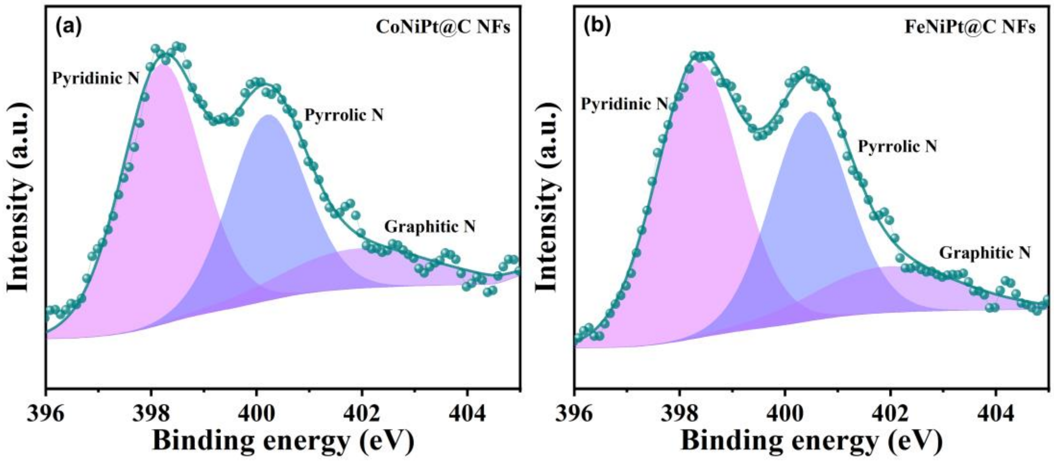

Figure S5. The high-resolution N 1s XPS spectra of (a) CoNiPt@C NFs and (b) FeNiPt@C NFs catalyst.

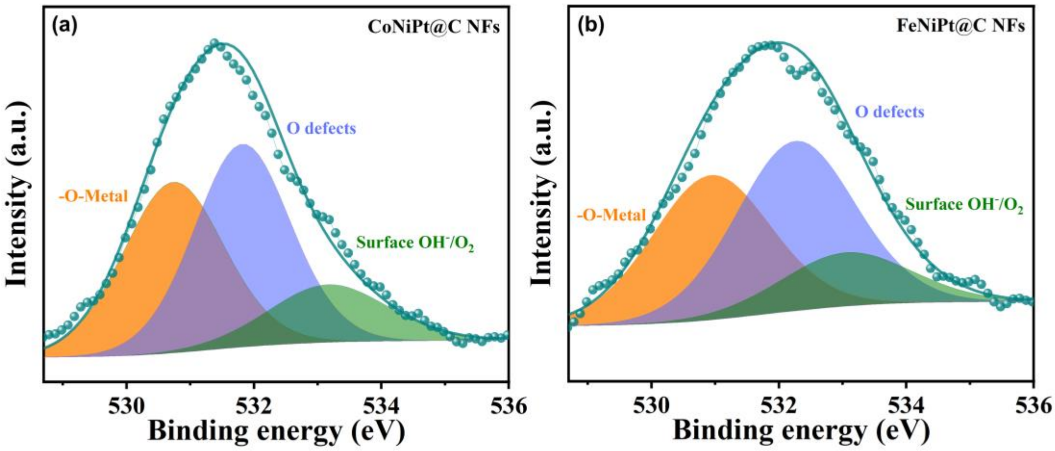

Figure S6. The high-resolution O 1s XPS spectra of (a) CoNiPt@C NFs and (b) FeNiPt@C NFs catalyst.

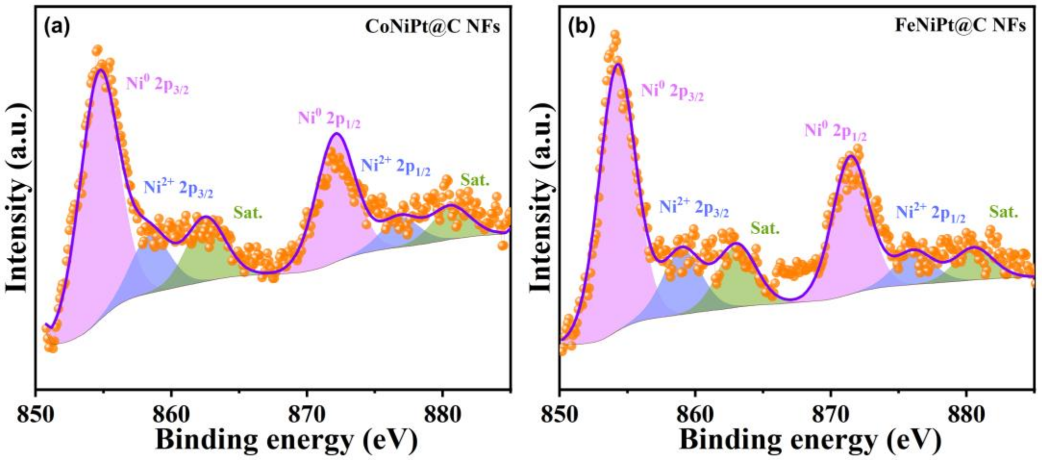

Figure S7. The high-resolution Ni 2p XPS spectra of (a) CoNiPt@C NFs and (b) FeNiPt@C NFs catalyst.

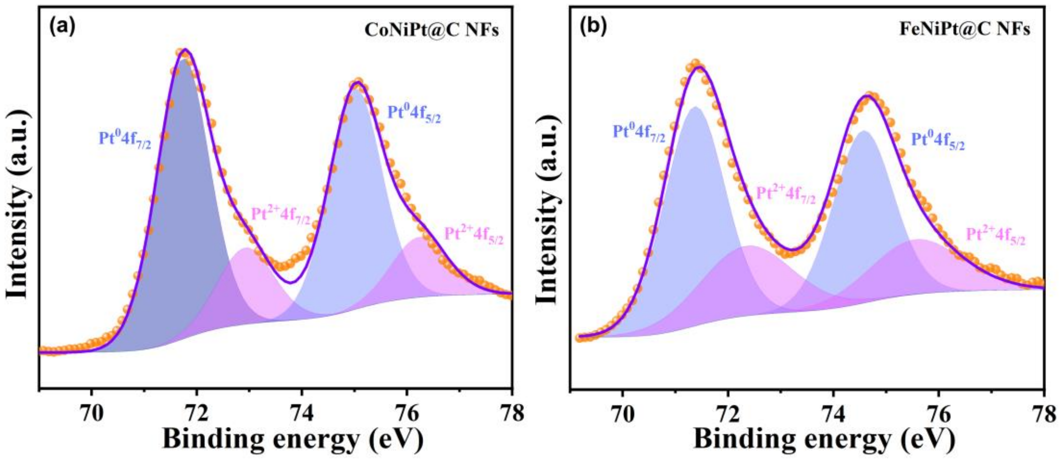

Figure S8. The high-resolution Pt 4f XPS spectra of (a) CoNiPt@C NFs and (b) FeNiPt@C NFs catalyst.

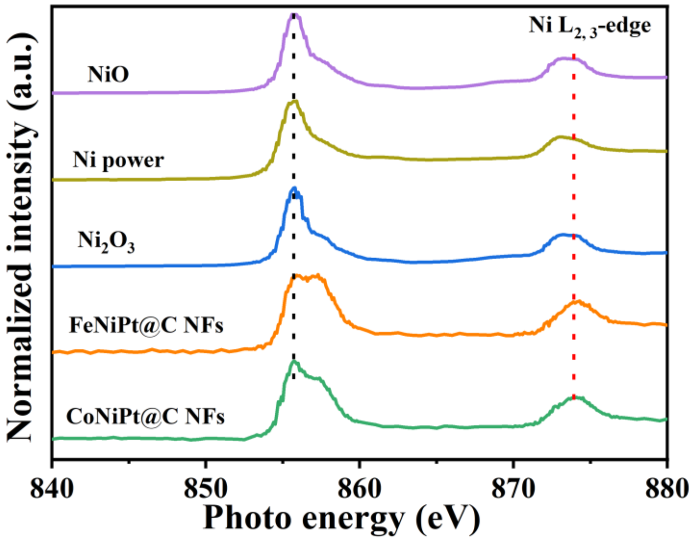

Figure S9. The Ni $L_{2,3}$-edge XANES spectra of FeNiPt@C NFs, CoNiPt@C NFs and standard samples.

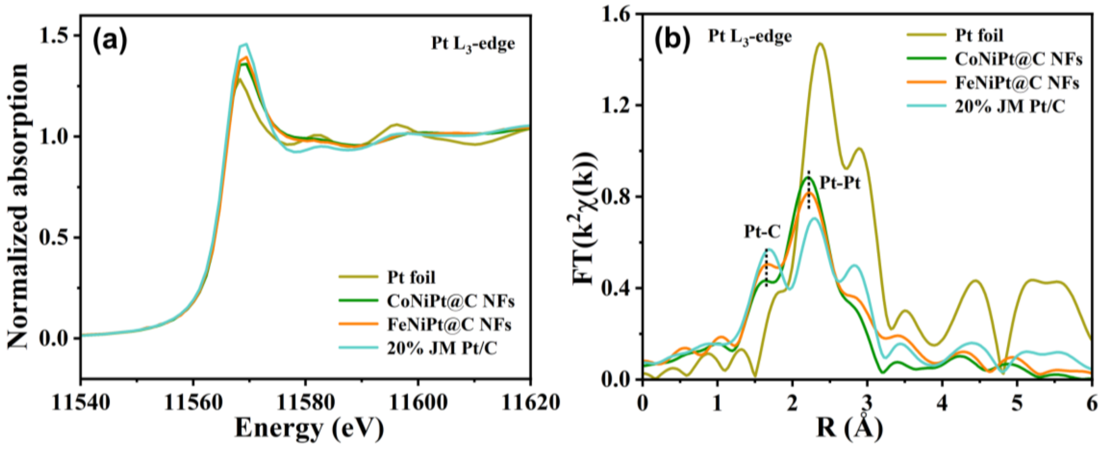

Figure S10. The XAFs spectra of the catalysts. (a) Pt L_3-edge XANES spectra. (b) The k^2-weight FT-EXAFS spectra of Pt L_3-edge.

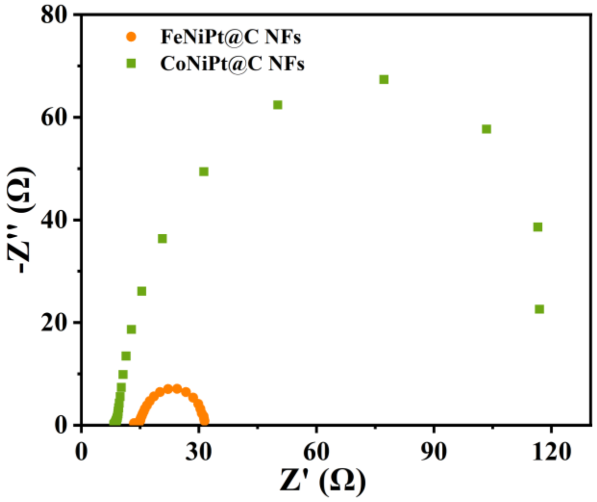

Figure S11. The EIS spectra of FeNiPt@C NFs and CoNiPt@C NFs for OER.

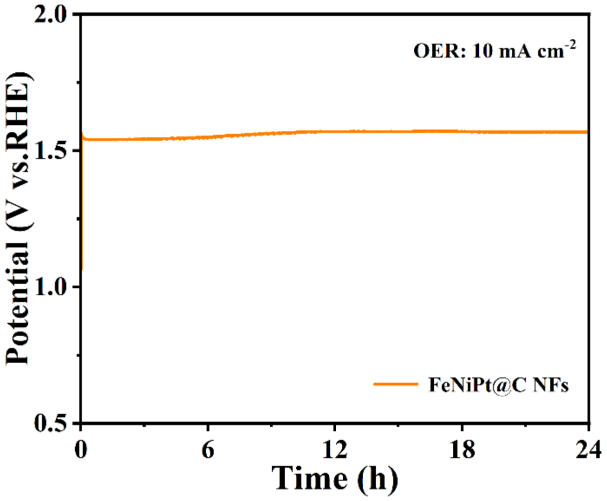

Figure S12. The OER chronopotentiometric curves at 10 mA cm^{-2} current density for FeNiPt@C NFs catalyst.

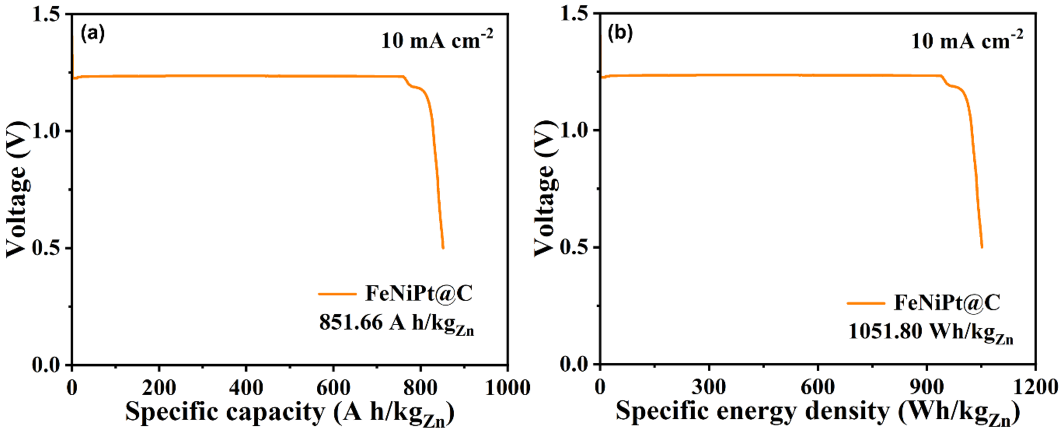

Figure S13. The galvanostatic discharge curves at 10 mA cm^{-2} current density of rechargeable ZAB with FeNiPt@C NFs catalyst. (a) Specific capacity, (b) Specific energy density.

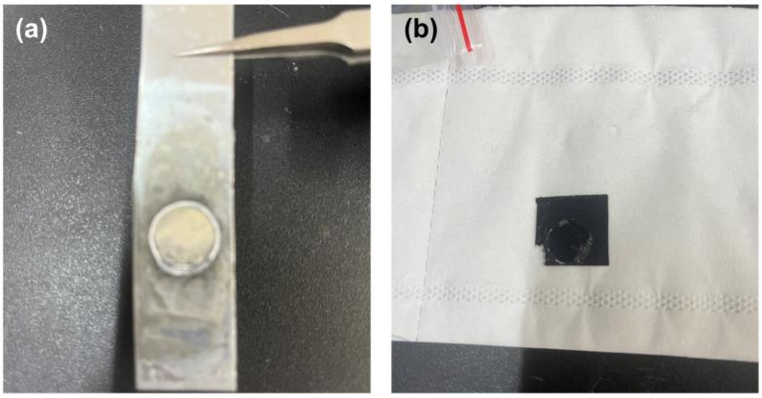

Figure S14. The optical photographs of (a) Zn negative electrode, (b) air eletrode coated with FeNiPt@C catalyst in ZAB after galvanostatic discharge-charge cycling curve.

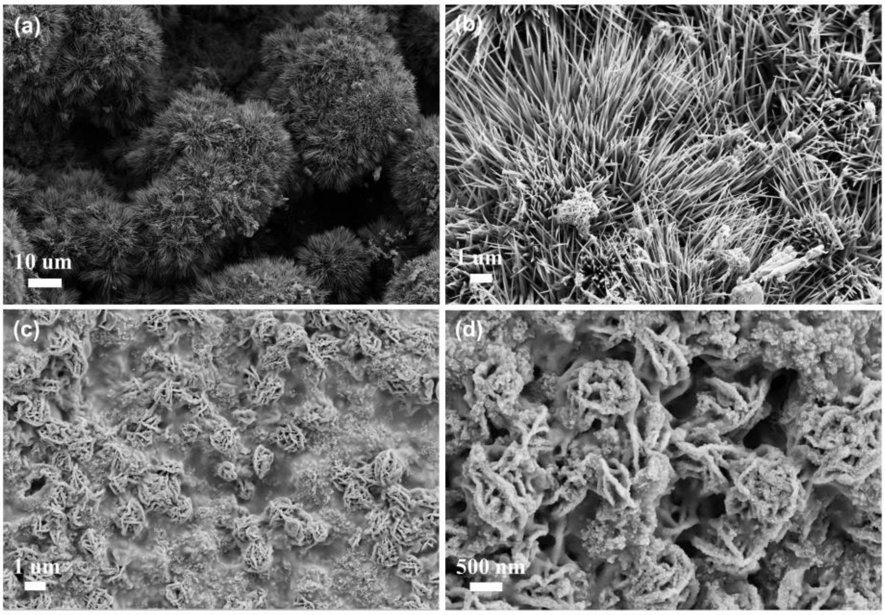

Figure S15. SEM images of Zn electrode (a-b) and air electrode (c-d) in FeNiPt@C ZAB after galvanostatic discharge-charge cycling curve.

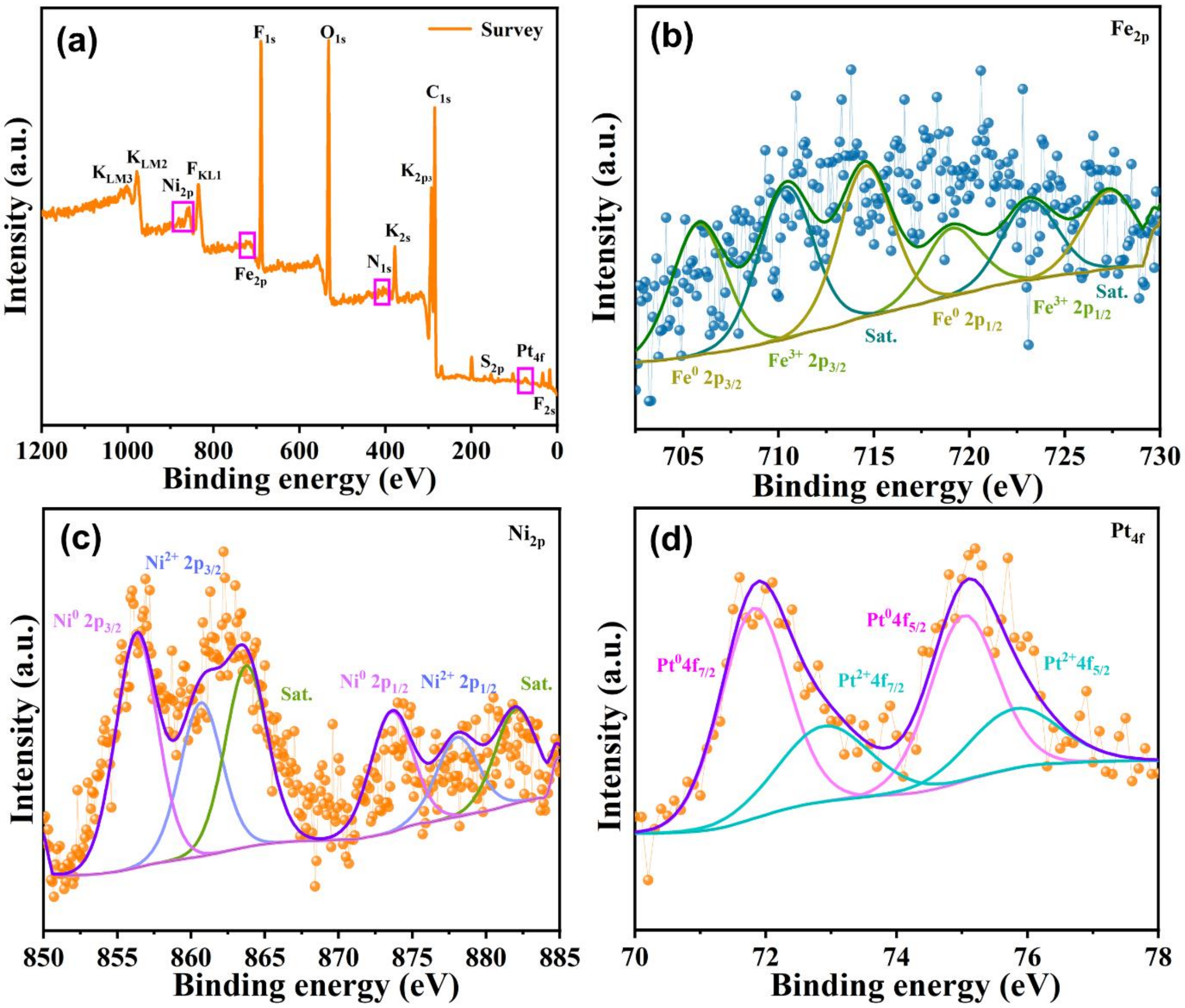

Figure S16. XPS spectra of air electrode in FeNiPt@C ZAB after galvanostatic discharge-charge cycling curve: (a) Survey spectrum. (b) High-resolution Fe 2p XPS spectrum. (c) High-resolution Ni 2p XPS spectrum. (d) High-resolution Pt 4f XPS spectrum.

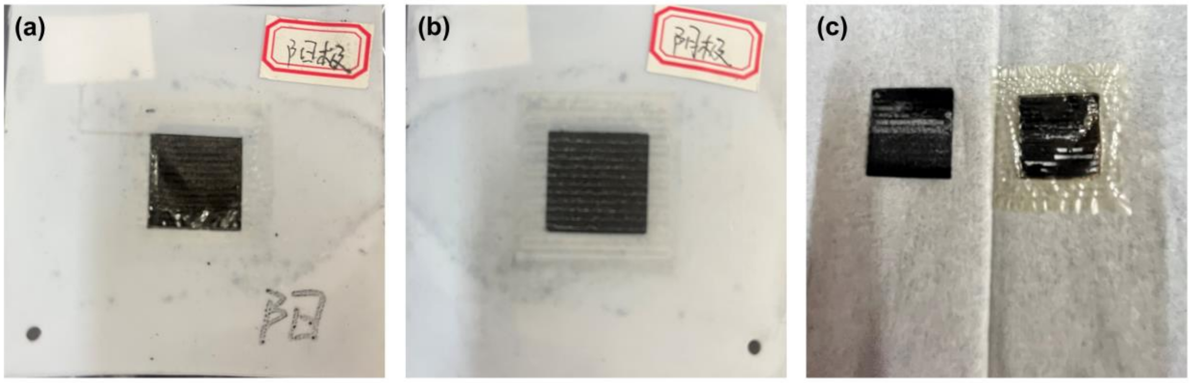

Figure S17. The diagram of in membrane electrode assembly (MEA) in AEMWE after chronopotentiometry test.

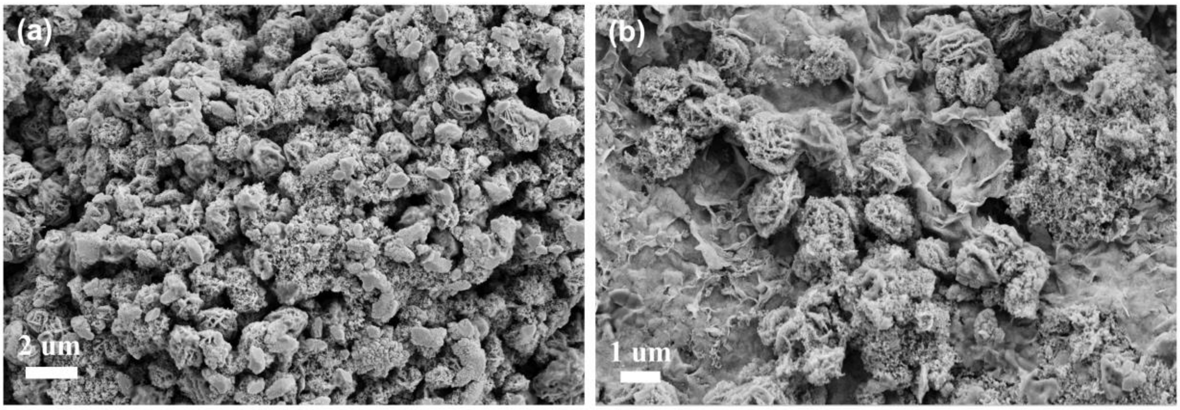

Figure S18. SEM images of cathode (a-b) in FeNiPt//FeNiPt AEMWE after chronopotentiometry test.

Table S1. The proportion of different N types of high-resolution N_{1s} XPS spectra.

Catalyst	Pyridinic N	Pyrrolic N	Graphitic N
FeNiPt@C NFs	50.1 %	35.4 %	14.4 %
CoNiPt@C NFs	50.8 %	34.7 %	14.5 %

Table S2. The proportion of different O types of high-resolution N_{1s} XPS spectra.

Catalyst	-O-Metal	O defects	Surface OH^-/O_2
FeNiPt@C NFs	38.8 %	45.6 %	15.6 %
CoNiPt@C NFs	39.4 %	44.8 %	15.8 %

Table S3. The fitting equivalent circuit diagrams of FeNiPt@C NFs and CoNiPt@C NFs.

Catalyst	Fitting equivalent circuit
FeNiPt@C NFs	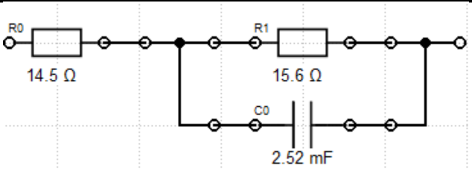
CoNiPt@C NFs	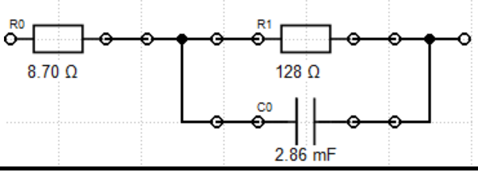

Table S4. The trifunctional HER/OER/ORR catalytic performance comparison of FeNiPt@C catalyst with other recently reported representative electrocatalysts.

Catalyst	OER/η_{10} (mV)	HER/η_{10} (mV)	ORR/$E_{1/2}$ (V)	Loading (mg/cm^{-2})	Ref.
FeNiPt@C NFs	294	25	0.93	0.25	This work
CoFeN-NCNTs//CCM	320	151	0.84	0.25 (ORR)<br>0.28 (OER)<br>0.28 (HER)	1
$Fe_{0.33}Sn_{0.67}OSe$/NF	162	61	0.84	--	2
Co_3W_3C/CoP/NPC	200	139	0.803	0.9	3
IrCo@NCNT/PC	240	37	0.83	0.5	4
CS-NFO@PNC-700	217	200	0.85	0.08 (ORR)<br>3.5 (HER)<br>3.5 (OER)	5
CoFe@NC/NCHNSs	285	120	0.92	0.25	6
Co-NC@CC	240	73	0.94	3	7
CMO-U@CC	309	194	0.81	--	8
Co-TMPyP/CCG	379	320	0.824	0.25	9
Mo_2C/Co@NC	308	51	0.95	0.3 (ORR)<br>0.5 (HER)<br>0.5 (HER)	10
Co/CoS/Fe-HSNC-700	250	138	0.906	0.25 (ORR)<br>1.2 (HER)<br>1.2 (OER)	11
Co_2P/CoNPC	326	208	0.843	0.39	12
CoPx@CNS/NF	286	91	0.76	--	13
Ni@N-HCGHF	260	95	0.875	0.2 (ORR)<br>--	14
W_2N/WC	320	148.5	0.81	0.306	15
CoSA/N, S-HCS	306	165	0.85	0.12 (ORR)<br>1.5 (HER)<br>1.5 (OER)	16
Fe_3C-Co/NC	340	238	0.885	0.2 (ORR)<br>0.4 (OER)<br>0.3 (HER)	17
CoC/NFs (1000)	320	190	0.896	0.3	18
NOGB-800	400	220	0.84	0.4	19
0.4-Ru@NG-750	372	40	0.826	--	20
FeS/Fe_3C@N-S-C-800	570	450	0.87	0.3 (ORR)<br>0.6 (HER)<br>0.6 (OER)	21
Co_2P NC	280	75.5	0.839	0.2	22
NiCoP/CNF	268	130	0.82	0.32 (ORR)<br>0.69 (HER)<br>1.0 (OER)	23
PPy/FeTCPP/Co	340	270	0.86	0.3	24

Table S5. Performance comparison of Zn-air battery and water electrolysis assembled with trifunctional catalysts.

Catalyst	Power density in ZAB	Loading/ZAB type	Current density in water electrolysis	Loading/device	Ref.
FeNiPt@C NFs	168 mW cm^{-2}	1.6 mg cm^{-2}, liquid-state	698 mA cm^{-2} at 1.85 V	2.0 mg cm^{-2}/CP, anion exchange membrane water electrolyzer	This work
CoFeN-NCNTs//CCM	145	--,	100 mA cm^{-2}	0.25 mg cm^{-2}/CP ,	1

	mW cm^{-2}	liquid-state	at 1.8 V	overall water-splitting cell	
$Fe_{0.33}Sn_{0.67}OSe$/NF	153.96 mW cm^{-2}	--, quasi solid-state	100 mA cm^{-2} at 1.811 V	--, overall water-splitting cell	2
Co_3W_3C/CoP/NPC	205.5 mW cm^{-2}	2.0 mg cm^{-2}, liquid-state	83 mA cm^{-2} at 2.0 V	3.0 mg cm^{-2}/NF, overall water-splitting cell	3
IrCo@NCNT/PC	136.8 mW cm^{-2}	1.0 mg cm^{-2}, liquid-state	150 mA cm^{-2} at 1.7 V	1.0 mg cm^{-2}/CC, overall water-splitting cell	4
CS-NFO@PNC-700	130 mW cm^{-2}	1.0 mg cm^{-2}, liquid-state	100 mA cm^{-2} at 2.17 V	3.0 mg cm^{-2}/CP, overall water-splitting cell	5
CoFe@NC/NCHNSs	184 mW cm^{-2}	1.0 mg cm^{-2}, liquid-state	87 mA cm^{-2} at 2.0 V	--, overall water-splitting cell	6
Co-NC@CC	170 mW cm^{-2}	--, liquid-state	80 mA cm^{-2} at 2.06 V	--, overall water-splitting cell	7
CMO-U@CC	135 mW cm^{-2}	--, liquid-state	100 mA cm^{-2} at 1.8 V	--, overall water-splitting cell	8
Co-TMPyP/CCG	225.4 mW cm^{-2}	1.0 mg cm^{-2}, liquid-state	--	--	9
Mo_2C/Co@NC	187.9 mW cm^{-2}	--, liquid-state	30 mA cm^{-2} at 1.8 V	--, overall water-splitting cell	10
Co/CoS/Fe-HSNC-700	213 mW cm^{-2}	--	35 mA cm^{-2} at 1.8 V	1.2 mg cm^{-2}, overall water-splitting cell	11
Co_2P/CoNPC	116 mW cm^{-2}	0.5 mg cm^{-2}, liquid-state	200 mA cm^{-2} at 1.9 V	1.2 mg cm^{-2}, overall water-splitting cell	12
CoPx@CNS/NF	110 mW cm^{-2}	--, liquid-state	160 mA cm^{-2} at 1.9 V	--, overall water-splitting cell	13
Ni@N-HCGHF	117.1 mW cm^{-2}	--, liquid-state	100 mA cm^{-2} at 1.72 V	--, overall water-splitting cell	14
W_2N/WC	172 mW cm^{-2}	0.6 mg cm^{-2}, liquid-state	50 mA cm^{-2} at 1.8 V	--, overall water-splitting cell	15
CoSA/N, S-HCS	173.1 mW cm^{-2}	1.5 mg cm^{-2}, liquid-state	200 mA cm^{-2} at 1.78 V	1.5 mg cm^{-2}/CC, overall water-splitting cell	16
Fe_3C-Co/NC	--	--	--	--	17
CoC/NFs (1000)	--	1.0 mg cm^{-2}, liquid-state	40 mA cm^{-2} at 1.8 V	--, overall water-splitting cell	18
NOGB-800	111.9 mW cm^{-2}	1.0 mg cm^{-2}, liquid-state	80 mA cm^{-2} at 2.0 V	--, overall water-splitting cell	19
0.4-Ru@NG-750	--	--	--	--	20
FeS/Fe_3C@N-S-C-800	63 mW cm^{-2}	4.0 mg cm^{-2}, liquid-state	--	--	21
Co_2P NC	62 mW cm^{-2}	0.5 mg cm^{-2}, button battery	80 mA cm^{-2} at 1.75 V	--, overall water-splitting cell	22
NiCoP/CNF	--	--	88 mA cm^{-2} at 2.0 V	--, overall water-splitting cell	23
PPy/FeTCPP/Co	--	0.5 mg cm^{-2}, liquid-state	10 mA cm^{-2} at 1.875 V	0.5 mg cm^{-2}, overall water-splitting cell	24

References

1. G. Zhou, G. Liu, X. Liu, Q. Yu, H. Mao, Z. Xiao and L. Wang, *Adv. Funct. Mater.* **2022**, *32*, 2107608.
2. K. Harish, J. Balamurugan, T. T. Nguyen, N. H. Kim and J. H. Lee, *Appl. Catal. B: Environ.* **2022**, *305*, 120924.
3. Y. Zhang, W. Shi, L. Bo, Y. Shen, X. Ji, L. Xia, X. Guan, Y. Wang and J. Tong, *Chem. Eng. J.* **2022**, *431*, 134188.
4. D. Zhao, Y. Zhu, Q. Wu, W. Zhou, J. Dan, H. Zhu, W. Lei, L.-J. Ma and L. Li, *Chem. Eng. J.* **2022**, *430*, 132825.
5. S. Ramakrishnan, D. B. Velusamy, S. Sengodan, G. Nagaraju, D. H. Kim, A. R. Kim and D. J. Yoo, *Appl. Catal. B: Environ.* **2022**, *300*, 120752.
6. S. Wang, H. Wang, C. Huang, P. Ye, X. Luo, J. Ning, Y. Zhong and Y. Hu, *Appl. Catal. B: Environ.* **2021**, *298*, 120512.
7. Y. Zhong, Y. Lu, Z. Pan, J. Yang, G. Du, J. Chen, Q. Zhang, H. Zhou, J. Wang, C. Wang and W. Li, *Adv. Funct. Mater.* **2021**, *31*, 2009853.
8. G. Janani, S. Surendran, H. Choi, M.-K. Han and U. Sim, Small **2021**, *17*, 2103613.
9. K. Cui, Q. Wang, Z. Bian, G. Wang and Y. Xu, *Adv. Energy Mater.* **2021**, *11*, 2102062.
10. T. Gu, R. Sa, L. Zhang, D.-S. Li and R. Wang, *Appl. Catal. B: Environ.* **2021**, *296*, 120360.
11. L. Yan, H. Wang, J. Shen, J. Ning, Y. Zhong and Y. Hu, *Chem. Eng. J.* **2021**, *403*, 126385.
12. H. Liu, J. Guan, S. Yang, Y. Yu, R. Shao, Z. Zhang, M. Dou, F. Wang and Q. Xu, *Adv. Mater.* **2020**, *32*, 2003649.
13. C.-C. Hou, L. Zou, Y. Wang and Q. Xu, *Angew. Chem. Int. Ed.* **2020**, *59*, 21360-21366.
14. L. Yan, Y. Xu, P. Chen, S. Zhang, H. Jiang, L. Yang, Y. Wang, L. Zhang, J. Shen, X. Zhao and L. Wang, *Adv. Mater.* **2020**, *32*, 2003313.
15. J. Diao, Y. Qiu, S. Liu, W. Wang, K. Chen, H. Li, W. Yuan, Y. Qu and X. Guo, *Adv. Mater.* **2020**, *32*, 1905679.
16. Z. Zhang, X. Zhao, S. Xi, L. Zhang, Z. Chen, Z. Zeng, M. Huang, H. Yang, B. Liu, S. J. Pennycook and P. Chen, *Adv. Energy Mater.* **2020**, *10*, 2002896.
17. C. C. Yang, S. F. Zai, Y. T. Zhou, L. Du and Q. Jiang, *Adv. Funct. Mater.* **2019**, *29*, 1901949.
18. Z. Yang, C. Zhao, Y. Qu, H. Zhou, F. Zhou, J. Wang, Y. Wu and Y. Li, *Adv. Mater.* **2019**, *31*, 1808043.
19. Q. Hu, G. Li, G. Li, X. Liu, B. Zhu, X. Chai, Q. Zhang, J. Liu and C. He, *Adv. Energy Mater.* **2019**, *9*, 1803867.

20. L. Bai, Z. Duan, X. Wen, R. Si, Q. Zhang and J. Guan, *ACS Catal*. **2019**, *9*, 9897-9904.
21. F. Kong, X. Fan, A. Kong, Z. Zhou, X. Zhang and Y. Shan, *Adv. Funct. Mater*. **2018**, *28*, 1803973.
22. H. Li, Q. Li, P. Wen, T. B. Williams, S. Adhikari, C. Dun, C. Lu, D. Itanze, L. Jiang, D. L. Carroll, G. L. Donati, P. M. Lundin, Y. Qiu and S. M. Geyer, *Adv. Mater*. **2018**, *30*, 1705796.
23. S. Surendran, S. Shanmugapriya, A. Sivanantham, S. Shanmugam and R. K. Selvan, *Adv. Energy Mater*. **2018**, *8*, 1800555.
24. J. Yang, X. Wang, B. Li, L. Ma, L. Shi, Y. Xiong and H. Xu, *Adv. Funct. Mater*. **2017**, *27*, 1606497.
